# MR Tractography-Based Targeting and Physiological Identification of the Cuneiform Nucleus for Directional DBS in a Parkinson’s Disease Patient With Levodopa-Resistant Freezing of Gait

**DOI:** 10.3389/fnhum.2021.676755

**Published:** 2021-06-08

**Authors:** Stephano J. Chang, Iahn Cajigas, James D. Guest, Brian R. Noga, Eva Widerström-Noga, Ihtsham Haq, Letitia Fisher, Corneliu C. Luca, Jonathan R. Jagid

**Affiliations:** ^1^The Miami Project to Cure Paralysis, Miami, FL, United States; ^2^Department of Neurosurgery, University of British Columbia, Vancouver, BC, Canada; ^3^Department of Neurological Surgery, University of Miami Miller School of Medicine, Miami, FL, United States; ^4^Department of Neurology, University of Miami Miller School of Medicine, Miami, FL, United States

**Keywords:** freezing of gait, gait dysfunction, Parkinson’s Disease, mesencephalic locomotor region, cuneiform nucleus, pedunculopontine nucleus

## Abstract

**Background:**

Freezing of gait (FOG) is a debilitating motor deficit in a subset of Parkinson’s Disease (PD) patients that is poorly responsive to levodopa or deep brain stimulation (DBS) of established PD targets. The proposal of a DBS target in the midbrain, known as the pedunculopontine nucleus (PPN), to address FOG was based on its observed neuropathology in PD and its hypothesized involvement in locomotor control as a part of the mesencephalic locomotor region (MLR). Initial reports of PPN DBS were met with enthusiasm; however, subsequent studies reported mixed results. A closer review of the MLR basic science literature, suggests that the closely related cuneiform nucleus (CnF), dorsal to the PPN, may be a superior site to promote gait. Although suspected to have a conserved role in the control of gait in humans, deliberate stimulation of a homolog to the CnF in humans using directional DBS electrodes has not been attempted.

**Methods:**

As part of an open-label Phase 1 clinical study, one PD patient with predominantly axial symptoms and severe FOG refractory to levodopa therapy was implanted with directional DBS electrodes (Boston Science Vercise Cartesia^TM^) targeting the CnF bilaterally. Since the CnF is a poorly defined reticular nucleus, targeting was guided both by diffusion tensor imaging (DTI) tractography and anatomical landmarks. Intraoperative stimulation and microelectrode recordings were performed near the targets with leg EMG surface recordings in the subject.

**Results:**

Post-operative imaging revealed accurate targeting of both leads to the designated CnF. Intraoperative stimulation near the target at low thresholds in the awake patient evoked involuntary electromyography (EMG) oscillations in the legs with a peak power at the stimulation frequency, similar to observations with CnF DBS in animals. Oscillopsia was the primary side effect evoked at higher currents, especially when directed posterolaterally. Directional DBS could mitigate oscillopsia.

**Conclusion:**

DTI-based targeting and intraoperative stimulation to evoke limb EMG activity may be useful methods to help target the CnF accurately and safely in patients. Long term follow-up and detailed gait testing of patients undergoing CnF stimulation will be necessary to confirm the effects on FOG.

**Clinical Trial Registration:**

Clinicaltrials.gov identifier: NCT04218526.

## Introduction

Gait disturbances feature prominently in Parkinson’s disease (PD) and contribute significantly to patient disability, a decreased quality of life, and increased morbidity through risk of falls (Kerr et al., 2010; Allen et al., 2013). Freezing of gait (FOG) is one of the most debilitating of these deficits, and is described as the sudden and paroxysmal inability to generate effective stepping, despite the intention to do so (Giladi and Nieuwboer, 2008). It is a poorly understood phenomenon without a single unifying pathology and may represent a heterogeneous collection of circuitopathies affecting nodes along the locomotor control network (Rahimpour et al., 2020). Perhaps consequently, the management of FOG is complicated by its variable response to dopaminergic therapy—while some patients improve with medication, others have freezing that is refractory to levodopa (Nonnekes et al., 2015). Further still, in a small subset of patients, FOG appears to be induced or exacerbated by dopaminergic treatments (Espay et al., 2012). Patients with FOG that does not improve with levodopa are considered poor candidates for deep brain stimulation (DBS) surgery targeting the usual subthalamic nucleus (STN) or globus pallidus interna (GPi) targets (Thevathasan et al., 2018), leaving this population with few viable treatment options.

**FIGURE 1:**
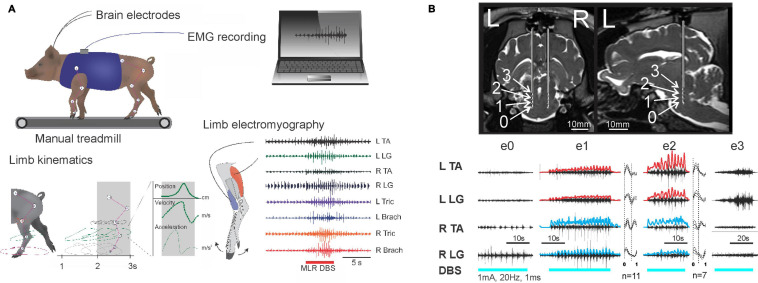
Electrical mapping of the MLR in a large animal model. **(A)** Experimental schematic in the micropig model. **(B)** Intraoperative stimulation of the MLR. Top: Coronal and sagittal views showing calculated positions of electrodes 0–3. Bottom: EMG responses to cathodic biphasic stimulation of electrode 0–3 on left side (1 mA, 20 Hz, and 1 ms). Rectified and high pass filtered traces of individual EMG traces from e1 and e2 are overlaid in red (left) and blue (right). Step cycle averages for e1 and e2 are shown on right of each muscle, with the number of step cycles averaged indicated. Best locomotor-like response is observed with e1 and e2 stimulation, located within the cuneiform and adjacent subcuneiform region. Adapted from Noga et al. (2020), with permission.

The mesencephalic locomotor region (MLR) has been identified as an important locomotor control center in the midbrain of multiple vertebrate species (Shik et al., 1966; Eidelberg et al., 1981; Skinner and Garcia-Rill, 1984; Cabelguen et al., 2003; Takakusaki et al., 2003). Functional imaging studies suggest that a homologous entity also exists in humans (Jahn et al., 2008), and clinicians have pursued this region as a potential DBS target to ameliorate gait dysfunction and FOG over the past 15 years with mixed reported outcomes (Mazzone et al., 2005; Plaha and Gill, 2005; Ferraye et al., 2010; Yeh et al., 2010; Thevathasan et al., 2011; Welter et al., 2015; Mestre et al., 2016). While animal studies have long distinguished between the pedunculopontine nucleus (PPN) and the slightly dorsally positioned cuneiform nucleus (CnF) in debates over the exact structural correlate to the MLR [(see Ferreira-Pinto et al. (2018) for a review], with many suggesting the CnF may be more efficacious for gait (Shik et al., 1966; Eidelberg et al., 1981; Takakusaki et al., 2016; Opris et al., 2019; Chang et al., 2021; Figure 1), neurosurgeons have exclusively targeted the PPN. This raises the possibility that target optimization in this region, including with the use of new directional DBS electrodes, could improve outcomes (Chang et al., 2020b).

Recently, several optogenetic studies targeting the MLR in mice have functionally characterized and distinguished neuronal populations within the MLR by neurochemistry and anatomy, suggesting that glutamatergic CnF neurons are the principal group within the MLR involved in initiating and controlling locomotion (Caggiano et al., 2018; Josset et al., 2018; Dautan et al., 2020). Supported by a recent anatomical-clinical study (Goetz et al., 2019), we hypothesized that targeting the CnF could lead to improved outcomes for PD patients with FOG and devised a pilot feasibility study. In this paper, we report our method of targeting the CnF using pre-operative DTI along with intraoperative physiology and preliminary post-surgical assessments of the effects of CnF DBS on gait in our first patient.

## Materials and Methods

The subject was recruited for this study from the Movement Disorders Clinic at the University of Miami Hospital. This study was approved by the University of Miami Human Subject Research Office (UM HSRO; IRB #20190702) and the United States Food and Drug Administration with an Investigational Device Exemption (G190164) as a phase I clinical trial. The trial is registered in ClinicalTrials.gov (NCT04218526), and the full study protocol is described elsewhere (Chang et al., 2020a). As part of the study protocol, the subject underwent a thorough multi-disciplinary evaluation, including psychological assessment and evaluation by multiple movement disorder specialists and a neurosurgeon, to determine surgical candidacy.

### Pre-operative Imaging and Planning

**FIGURE 2:**
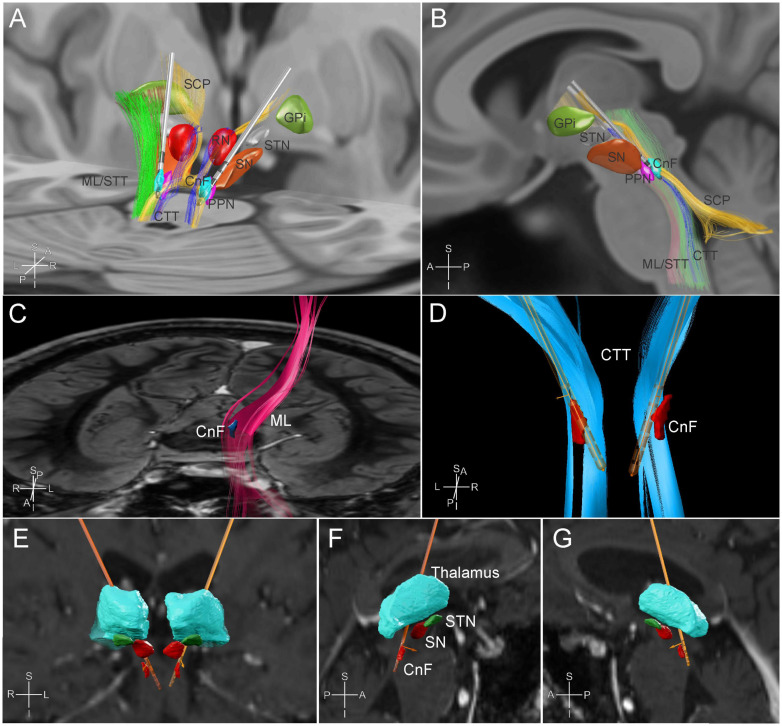
MR tractography-based targeting of the cuneiform nucleus. Posterior oblique **(A)** and sagittal **(B)** views of a three-dimensional reconstruction of the regional anatomy and tractography based on available template atlases in Lead DBS (Horn and Kühn, 2015; Ewert et al., 2018; Yeh et al., 2018). A model of the Boston Scientific Vercise Cartesia^TM^ directional electrode is placed in the field targeting the cuneiform nucleus bilaterally for demonstration. CnF, cuneiform nucleus; CTT, central tegmental tract; GPi, globus pallidus internus; ML, medial lemniscus; PPN, pedunculopontine nucleus; RN, red nucleus; SCP, superior cerebellar peduncle tracts; SN, substantia nigra; STN, subthalamic nucleus; STT, spinothalamic tract. **(C–G)** Subject specific tractography-based targeting, visualized in Brainlab Elements software (Brainlab AG, Munich, Germany). **(C)** Frontal view from above of subject’s left medial lemniscus reconstruction (fuchsia) in relation to a preplanned estimate of the CnF target (blue) against a pons level axial slice of the brain. **(D)** Posterior view of the final electrode positions in relation to the estimated CnF target (red) and the subject’s reconstructed central tegmental tracts (light blue). **(E)** Frontal and **(F,G)** sagittal views of the final electrode positions in relation to the thalamus, substantia nigra (SN), subthalamic nuclei (STN), and CnF (red).

The subject underwent pre-operative imaging as an outpatient 1 week prior to DBS surgery, including multi-planar, multi-echo 3T MRI sequences with and without contrast, as well as diffusion tensor imaging (DTI) using a Siemens Magneton Vida 3T with a standard Siemens 32 channel head coil (Siemens, Erlangen, Germany). T1 imaging was acquired with the following sequence parameters: repetition time (TR) = 1,900 ms; echo time (TE) = 4.9 ms, matrix = 256 × 206; field of view (FOV) = 200 mm × 250 mm; slice thickness = 1.30 mm; scan time 11 min, 24 s. T2 imaging was acquired with the following sequence parameters: TR = 4,780 ms; TE = 79 ms; matrix = 256 × 256; FOV = 250 mm × 250 mm; slice thickness = 2.00 mm; scan time = 3 min, 49 s. The DTI was acquired with the following sequence parameters: TR = 5,300 ms, TE = 75 ms; matrix 128 × 128; FOV = 250 mm × 250 mm; slice thickness = 4.00 mm; scan time 8 min, 19 s. The DTI was non-linearly transformed to the structural T1 MRI to correct for susceptibility-induced and eddy current-induced distortions using the Brainlab cranial distortion correction algorithm and then used to estimate the tractography of the superior cerebellar peduncle [SCP; seeds = dentate nucleus, red nucleus; fractional anisotropy (FA) = 0.15; minimum length = 80 mm; maximum angle = 20°], the medial lemniscus (ML; seeds = posterior to medullary pyramids, ventroposterolateral nucleus of thalamus; FA = 0.15; minimum length = 80 mm; maximum angle = 20°), and the central tegmental tract (CTT; seeds = red nucleus, inferior olivary nucleus; FA = 0.08; minimum length = 54 mm; maximum angle = 20°) using Brianlab Elements Fibertracking (Brainlab AG, Munich, Germany), based on fiber assignment by continuous tracking. Since the cuneiform nucleus is not visible on MRI, three-dimensional reconstructions of these tracts were superimposed onto the T1 MRI to guide our targeting of the CnF based on its known relationships to these tracts (Figure 2).

At the level of the pontomesencephalic junction, starting with brainstem normalized coordinates (0.66, 0.4, 0), the target was adjusted posteriorly to accommodate a point that was posteromedial to the ML and posterolateral to the CTT and SCP [see (Goetz et al., 2019) for a review of the brainstem normalized coordinate system]. Once the tractography-guided target had been selected, the images were imported into the Medtronic Stealth Planning Station (Medtronic, Minneapolis, MN, United States) to calculate AC-PC coordinates for the targets (Right: Lateral +2.79 mm, AP −20.71 mm, dorsoventral −13.95 mm, with AC-PC distance of 28.1 mm; Left: −2.47 mm, AP −20.08 mm, dorsoventral −13.94 mm) and to create appropriate electrode trajectories avoiding cortical sulci, vessels, and ventricles. Additionally, the trajectories were planned to place the six directional contacts of each lead (contacts 2–7) at the estimated CnF target.

### Surgical Implantation and Intraoperative Physiology

Surgery was performed similarly to standard DBS cases reported previously (Okun et al., 2012; Cordeiro et al., 2020; Vitek et al., 2020). Local infiltration and light sedation were used to fix the Cosman–Roberts–Wells (CRW) head frame (Integra Life Sciences, Plainsboro, NJ, United States) to the subject’s skull, and a CT scan was performed to co-register the frame to the pre-operative imaging. In addition to the standard intraoperative monitoring for DBS implantation, differential surface electromyography (EMG) electrodes were placed over the subject’s *rectus femoris* (RF), *medial gastrocnemius* (MG), *biceps femoris* (BF), and *tibialis anterior* (TA) bilaterally. This was done to detect if stimulation near the predicted CnF would elicit limb EMG activity, as has been reported in animal studies. A video camera was used to record leg movements.

With the subject awake, off medication, and under tight blood pressure control (targeting a systolic blood pressure of 100–120 mmHg), microelectrode recordings (MERs) were performed along the planned trajectory using a single microelectrode in the Ben-Gun array (NeuroNav, Alpha Omega Co., Alpharetta, GA, United States). Beginning at +3 mm from the planned target, stimulation was performed along the trajectory while recording leg EMGs and observing for off-target effects (stimulation parameters: frequency, 20 Hz; pulse width, 100 μs; current amplitude, 0.1–2 mA). Involuntary leg EMG oscillations were observed with stimulation near our target at currents between 0.6–2 mA. The most common side effect of stimulation reported by the subject was oscillopsia, occurring reproducibly in this region at 1.5–2.0 mA. Based on our intraoperative physiology, an octopolar directional DBS electrode (Vercise Cartesia^TM^, Boston Scientific) was implanted to center the directional electrodes at the region that best elicited leg EMG oscillations. The same procedure was repeated for the left side, again with careful monitoring and control of blood pressure. After implantation of both electrodes, the patient was placed under general anesthesia, and the sub-clavicular generator and extension cables were placed to connect to the implanted leads.

### Post-operative Management

The subject received a post-operative CT scan (1 mm slices) to identify electrode positions and to rule out hemorrhage. Systolic blood pressure control was relaxed to 140 mmHg and the subject was admitted overnight for monitoring. The subject was discharged the following day and scheduled for a clinic follow up visit 2 weeks after surgery for DBS programming by CL, IH, BN, and SC.

### LFP Signal Processing

Local field potential (LFP) data captured using the NeuroSmart system (Alpha Omega Co., Alpharetta, GA, United States) were processed using MATLAB 9.9 R2020b (The Mathworks, Natwick, MA, United States) with custom written scripts. LFP data was sampled at 760 Hz and band-pass filtered between 1 and 250 Hz. Spectrograms were calculated using a short-time Fourier transform with 300 ms Kaiser windows and 50% overlap (Matlab function spectrogram) for each depth recorded near the target while the patient was at rest.

Spectral-depth maps were created by first computing the power spectral density from 1 to 30 Hz using the multi-taper method using slepian tapers (Matlab pmtm function) at each recorded depth. The spectral densities were then aggregated into a matrix (with one column for each depth recorded) and data used to construct an image (Matlab imagesc function).

### EMG Preprocessing and Feature Extraction

Surface EMGs were collected intraoperatively as European Data Format (.edf) files sampled at 256 Hz and pre-processed with a high-pass filter above 10 Hz using a 7th order Chebyshev filter in EDFbrowser (De Luca, 2003). These signals were then loaded into MATLAB 9.9 R2020b (The Mathworks, Inc., Natick, MA, United States) for feature extraction and analysis (Too et al., 2019). EMG characteristics analyzed included measures of amplitude (mean absolute value, enhanced mean absolute value, root-mean-square) and frequency (zero crossing, slope sign change SSC). The right *rectus femoris* was analyzed as a representative muscle in this preliminary study. An EMG spectral-depth map was created using the same method described for the LFPs, but for frequencies between 0 and 50 Hz.

### Gait Assessments

For the Timed up and go test, the total time required for the subject to stand up from a chair, walk forward 3 m, turn around, walk back to the chair, and sit down, was recorded. For the turning tests, the patient made a complete 360° turn on the spot in either the clockwise or counterclockwise direction. The total time and the number of steps were recorded. For calculation of gait parameters, a 2-min walk test with turns was performed by the subject wearing Opal inertial measurement unit sensors (APDM Inc., Portland, OR, United States). Gait parameters were calculated using the Opal software (APDM, Inc., Portland, OR, United States) and include stride length and velocity, gait cycle time and cadence, percent of step cycle spent in swing vs. stance, arm and shank range of movement, turning time and the number of steps per turn, and the phase coordination index—a measure of bilateral coordination (Plotnik et al., 2007). Measures of gait variability were calculated from the individual gait cycle times and cadences.

### Data Analysis

For all tests, an α threshold of 0.05 was set to determine statistical significance. RStudio was used to perform statistical analyses. Differences between the DBS ON and DBS OFF state were compared using a paired *t*-test.

## Results

As the nuclei comprising the MLR are reticular structures not easily visible with available MR sequences, surgeons have relied on the use of coordinate systems and anatomical landmarks to target these regions. Some surgeons have thus resorted to using a brainstem normalized coordinate system to account for individual differences in anatomy that might affect accurate targeting of this area (Goetz et al., 2019). Diffusion weighted imaging performed in our subject allowed us to estimate the tractography of known tracts in this region to adjust our initial target coordinates for this specific subject (Figure 2). Post-operative imaging revealed accurate positioning of leads at the designated targets.

### Intraoperative LFP Recordings Near the Cuneiform Nucleus

**FIGURE 3:**
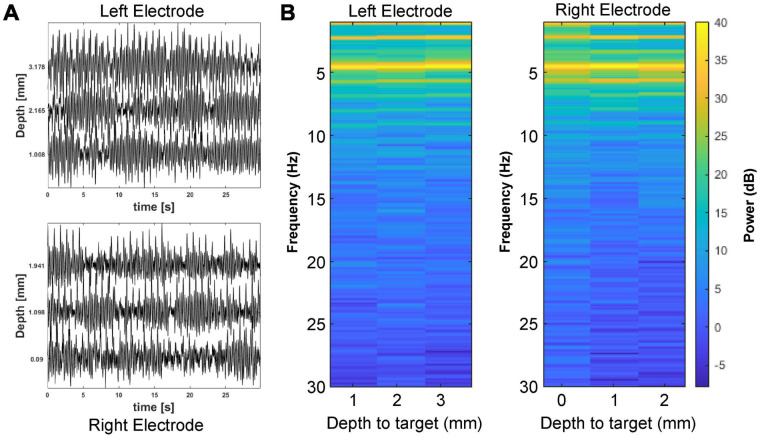
Local field potential (LFP) recordings near the cuneiform nucleus. **(A)** Baseline LFP recordings from the left and right brainstem are shown for several depths approaching the planned electrode tip target. **(B)** LFP spectrograms are plotted for the left and right electrodes by distance to the planned electrode tip target.

Local field potential recordings were made with electrodes on each side at multiple depths approaching the cuneiform nucleus target. Figure 3 shows the LFP recordings made with the microelectrode on each side during its advance to the planned electrode tip target, spanning the estimated location of the cuneiform nucleus. Recordings from this region with the subject at rest showed spectral power peaks in the theta range (4–8 Hz) bilaterally, common oscillatory frequencies that have been reported in association with the FOG phenomenon (Shimamoto et al., 2010; Chen et al., 2019; Marquez et al., 2020). Similar peaks in the beta (15–25 Hz) range were not observed.

### Leg EMG Changes With Stimulation Near the Cuneiform Nucleus

**FIGURE 4:**
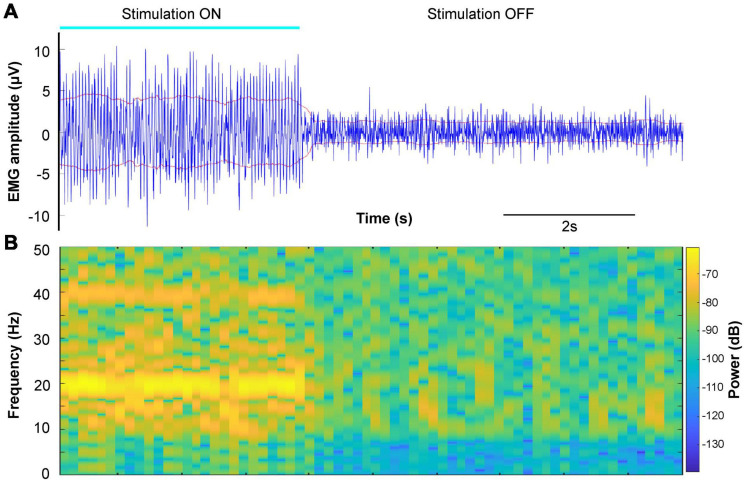
Example of surface EMG changes during DBS of the cuneiform nucleus target. **(A)** Right rectus femoris surface EMG (blue) during and after cessation of stimulation (1 mA, 20 Hz, and 0.2 ms) near the cuneiform nucleus target. The root-mean-square envelope for the signal is shown in red. **(B)** Spectrogram of the EMG signal in panel **(A)**.

In animal models, intraoperative electrical stimulation of the cuneiform nucleus elicits EMG activity and sometimes even visible limb movements, which can be used as a physiological biomarker for targeting this area (Takakusaki et al., 2016; Opris et al., 2019). Thus, we sought to see if similar responses could be elicited with electrical stimulation of this area in our subject (Figure 4). Stimulation of our estimated targets on both sides evoked involuntary EMG activity that was observed in each of the leg muscles recorded for the duration of stimulation, without gross leg movements. Spectral analysis demonstrated the presence of a strong peak at 20 Hz and multiples, the stimulation frequency (Figure 4B). This peak did not appear at lower amplitude stimulation, potentially suggesting a motor thresholding effect (Supplementary Figure 1). Table 1 shows that the analyzed EMG features extracted from multiple DBS-ON and DBS-OFF epochs were significantly changed during intraoperative DBS of this target.

**TABLE 1:** Changes in EMG features during intraoperative stimulation of the cuneiform nucleus.

**EMG feature**	**Stimulation OFF (mean ± SD)**	**Stimulation ON (mean ± SD)**	***P*-value**
Mean absolute value	0.96 ± 0.04	3.68 ± 0.29	0.0033
Enhanced mean absolute value	0.90 ± 0.02	2.21 ± 0.11	0.0017
Root-mean-square	1.24 ± 0.06	4.56 ± 0.37	0.0037
Zero crossing	553.7 ± 21.1	325.3 ± 8.3	0.0019
Slope sign change	583.0 ± 19.0	509.3 ± 37.4	0.032

*SD, standard deviation*

### Stimulation-Induced Side Effects

Stimulation at higher current amplitudes induced side effects intraoperatively and in the clinic. The most significant and least tolerable side effect was oscillopsia, where the subject reported seeing objects in his entire visual field shaking. This was reliably reproduced with stimulation above certain thresholds and did not appear to dissipate over time; no gross movement of the eyes was perceptible. Notably, directing stimulation anteriorly reduced this side effect and increased the current threshold for evoking it by almost double, while posteriorly directed stimulation enhanced this phenomenon. Decreasing stimulation pulse width from 0.2 to 0.1 ms also alleviated this side effect, allowing significant increases to the current amplitude before it was encountered again. Other side effects described included a feeling of nasopharyngeal or ear fullness, headache, and nausea, each of which was better tolerated by the subject and which partially resolved over time.

### Preliminary Results of Cuneiform Nucleus DBS in Freezing of Gait

The subject (male, 66 years old at surgery) was diagnosed with PD 6 years prior to surgery, with initial complaints of difficulty writing due to right-hand stiffness, which progressed to gait difficulties the following year. While he initially responded to levodopa therapy, his gait problems worsened to severe FOG resulting in falls and became refractory to levodopa medication. The subject’s issues are primarily axial in nature.

Implantation of DBS leads was uneventful and without surgical complications. Although the subject did continue to experience falls after surgery, neither he nor his family members considered these to be above his prior baseline. Table 2 compares the subject’s baseline gait and turning to his 6-week post-operative visit (after 4 weeks of DBS ON). Significant improvements were seen in the timed up and go and turning tests, except for counterclockwise turn time, which showed a trend toward improvement. Gait and turning parameters during the 2-min walk test with DBS showed significant improvements in stride length and velocity, with reductions in gait variability (as measured by gait cycle time and cadence) and phase coordination index (better bilateral coordination). Notably, turning time and the steps per turn decreased significantly (Supplementary Video 1).

**TABLE 2:** Preliminary gait testing results.

**Gait test**	**Baseline (mean ± SD)**	**DBS ON (4 weeks) (mean ± SD)**	***P*-value**
Timed up and go	27.6 ± 2.2 s	15.6 ± 1.7 s	0.026
CW 360° turn (time)	27.1 ± 4.1 s	7.9 ± 1.2 s	0.024
CW 360° turn (steps)	21.0 ± 4.4	7.7 ± 1.2	0.046
CCW 360° turn (time)	47.4 ± 18.0 s	12.5 ± 4.4 s	0.069
CCW 360° turn (steps)	37.7 ± 11.4	11.0 ± 1.7	0.041
Gait parameters			
Stride length (m)	1.04 ± 0.20	1.24 ± 0.05	1.2 × 10^–9^
Stride velocity (m/s)	0.685 ± 0.153	0.925 ± 0.079	1.5 × 10^–14^
Gait cycle time (s)	1.53 ± 0.24	1.35 ± 0.11	1.6 × 10^–6^
Gait cycle time variability	0.155	0.082	
Cadence (steps/min)	80.6 ± 16.3	89.5 ± 7.0	0.0002
Cadence variability	0.202	0.078	
Swing (%)	27.0 ± 3.9	32.7 ± 1.4	4.2 × 10^–14^
Stance (%)	73.0 ± 3.9	67.3 ± 1.4	4.2 × 10^–14^
Arm RoM (degrees)	19.1 ± 4.9	29.6 ± 6.8	<2.2 × 10^–16^
Shank RoM (degrees)	58.9 ± 10.4	66.8 ± 2.4	<2.2 × 10^–16^
Turning time (s)	9.4 ± 3.5	3.3 ± 0.5	0.0006
Steps per turn	12.3 ± 4.2	5.6 ± 0.7	0.001
Phase Coordination Index (%)	14.9	7.95	

*CW, clockwise; CCW, counterclockwise; RoM, range of movement; SD, standard deviation.*

## Discussion

Although numerous studies have targeted the MLR in PD patients over the past 15 years as a potential site for neuromodulation to alleviate gait deficits, few have looked to optimize target selection or electrode positions to maximize this effect. Furthermore, no other groups have used the more recently available directional electrode technology in this region, where the ability to steer current in specific directions could be helpful in minimizing the activation of unrelated fiber tracts passing through the region. Based on many electrical mapping studies (Shik et al., 1966; Eidelberg et al., 1981; Takakusaki et al., 2016; Opris et al., 2019; Chang et al., 2021), as well as more recent optogenetic studies of the MLR (Caggiano et al., 2018; Josset et al., 2018; Dautan et al., 2020), we proposed to target the cuneiform nucleus, rather than the traditionally targeted PPN.

We used the subject’s regional DTI tractography to refine our pre-determined brainstem normalized coordinates for this target, since this region does not have clear boundaries on T1- or T2-weighted MR imaging (Zrinzo et al., 2008; Shimamoto et al., 2010; Cong et al., 2018). LFP recordings near our target during rest demonstrated an obvious peak in the theta range, consistent with some prior literature in PD patients with gait freezing in the PPN (Shimamoto et al., 2010) and in the STN (Chen et al., 2019). We did not observe a peak in the beta range, as has been reported in some electrophysiological studies of the PPN (Weinberger et al., 2008; Shimamoto et al., 2010), although at least one PPN study did not find LFP peaks in the beta range (Androulidakis et al., 2008), with no clearly accepted physiological markers of the PPN during MER mapping (Molina et al., 2020). Taking a cue from animal studies of CnF stimulation, we recorded limb EMGs during stimulation of our target. In these studies, it has been demonstrated that MLR stimulation frequency controls locomotor speed and frequency of locomotor movements (Sirota et al., 2000; Cabelguen et al., 2003; Chang et al., 2021). The ability to evoke involuntary EMG oscillations in the subject’s legs with a power peak at the stimulation frequency was encouraging as a potential biomarker for the target and has not previously described in humans. Mechanistically, this may involve the activation of descending reticulospinal and monoaminergic pathways that are classically described as being controlled by the MLR in cats (Noga et al., 2003, 2017).

Stimulation near our predicted cuneiform target also elicited side effects at higher currents, with oscillopsia being the most prominent. Notably, this side effect has been described previously with PPN DBS (Ferraye et al., 2009; Jenkinson et al., 2012), with stimulation of the oculomotor nerve (Ferraye et al., 2009), the superior cerebellar peduncle and cerebellar uncinate fasciculus (Jenkinson et al., 2012), and the medial longitudinal fasciculus proposed as potential causes (Fournier-Gosselin et al., 2013). Based on our finding using directional electrodes that posteriorly directed stimulation enhanced this effect while anteromedially directed stimulation ameliorated it, involvement of the trochlear nerve as it courses posteriorly and laterally around the inferior colliculus appears to fit best with the regional anatomy, rather than the oculomotor nerve (superomedial), the superior cerebellar peduncle (medial), or the medial longitudinal fasciculus (medial). Our subject’s reported feeling of nasopharyngeal or ear fullness may relate to stimulation of the nearby mesencephalic nucleus of the trigeminal nerve, which is known to receive proprioceptive afferent input from the nose, palate, and teeth (Linden, 1978).

There are important limitations to our study. Due to the COVID-19 pandemic, we have currently implanted only one subject in our study, with only preliminary gait data. We also did not stimulate points outside of the CnF to definitively rule out DBS artifact in the EMG recordings, though stimulation at lower amplitudes suggested a motor threshold. Future subjects will help us determine if this is a true biomarker of the region or a DBS artifact. Furthermore, while our subject’s improvements in gait and turning parameters after DBS are promising, our pilot study is primarily designed to demonstrate safety and feasibility. Future, larger studies based on this one may be able to confirm efficacy in improving gait dysfunction associated with conditions such as PD, spinal cord injury, or stroke. Interestingly, our subject is able to reliably detect when stimulation is turned on, stating that his legs feel like they “want to go.” This unfortunately makes it difficult to blind the subject to DBS during gait testing to control for potential placebo effects, a known challenge in designing effective neuromodulation studies (Boakye et al., 2021).

## Conclusion

We describe the first implantation of directional electrodes in the human MLR and the first deliberate targeting of the more posteriorly located cuneiform nucleus in a subject with PD and refractory FOG, as part of a pilot study. Targeting was guided by the subject’s regional DTI tractography and intraoperative physiology suggested similarities to observations in animal studies of the cuneiform nucleus. Our study provides evidence that a functional homolog to the cuneiform nucleus exists in humans and can be safely targeted for DBS.

## Data Availability Statement

The raw data supporting the conclusions of this article will be made available by the authors, without undue reservation.

## Ethics Statement

The studies involving human participants were reviewed and approved by University of Miami Human Subject Research Office. The patients/participants provided their written informed consent to participate in this study.

## Author Contributions

JJ is the principal investigator and performed surgery. SC drafted the manuscript and created the Figures. SC, IC, and CL collected the data. SC and IC analyzed the data. All authors were involved in the conception and design of the study as well as the reviewing and editing of the manuscript and read and approved the final manuscript.

## Conflict of Interest

JJ and CL have consulting agreements with Medtronic, Boston Scientific, and Abbott Medical. JJ has two funded grants through Medtronic and Boston Scientific. The remaining authors declare that the research was conducted in the absence of any commercial or financial relationships that could be construed as a potential conflict of interest.

## Supplementary Material

Supplementary Figure 1 **(A)** EMG spectrogram from same representative muscle in Figure 4 during stimulation at 0.2 mA, 20 Hz, and 0.2 ms with no obvious power peak. **(B)** EMG spectrogram from the same muscle during stimulation at 0.4 mA, 20 Hz, and 0.2 ms, beginning to show a power peak at 20 Hz.

Supplementary Video 360° turn at pre-operative baseline and after surgery with and without CnF DBS.
