# Supplementary figures and images for: MR Tractography-Based Targeting and Physiological Identification of the Cuneiform Nucleus for Directional DBS in a Parkinson’s Disease Patient With Levodopa-Resistant Freezing of Gait
Source: Front Hum Neurosci. 2021 Jun 8;15:676755. doi: 10.3389/fnhum.2021.676755 (PMC8217631; doi:10.3389/fnhum.2021.676755)

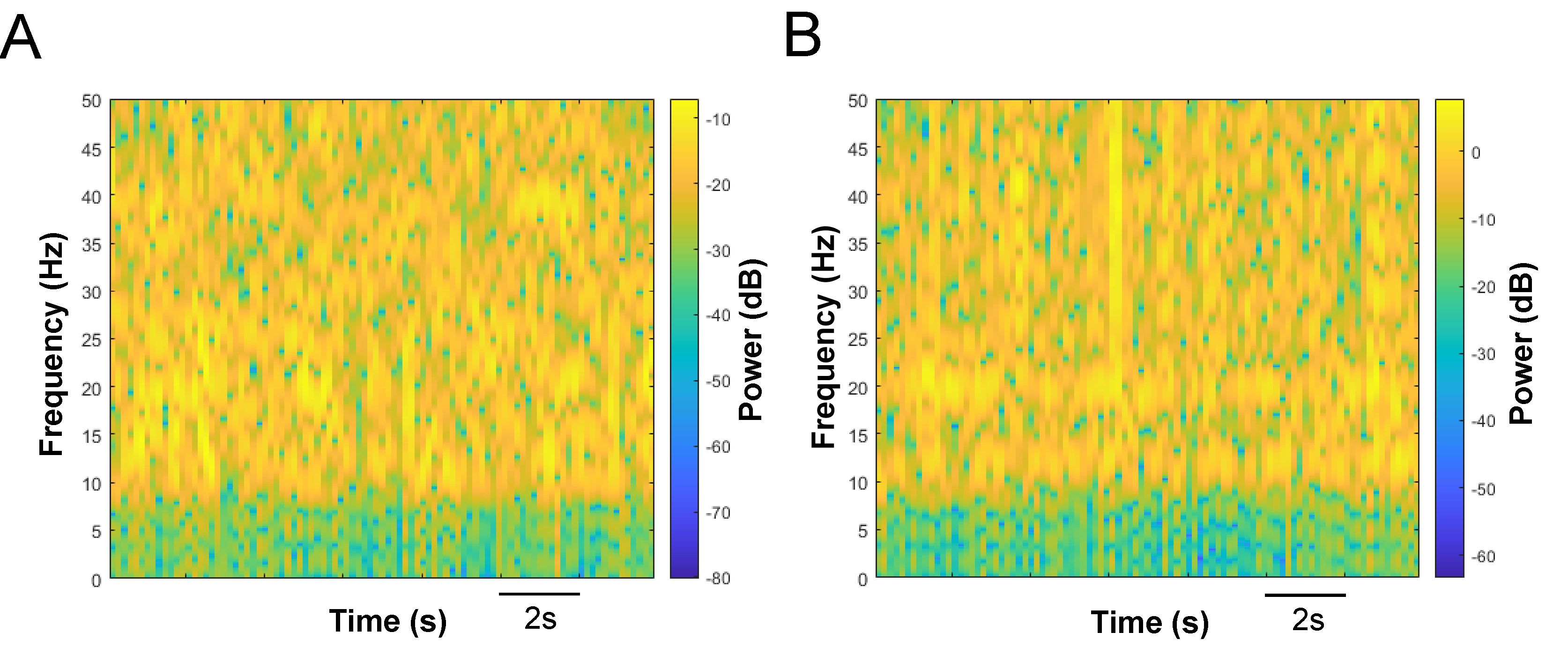

Supplement: Supplementary Figure 1 — (A) EMG spectrogram from same representative muscle in Figure 4 during stimulation at 0.2 mA, 20 Hz, and 0.2 ms with no obvious power peak. (B) EMG spectrogram from the same muscle during stimulation at 0.4 mA, 20 Hz, and 0.2 ms, beginning to show a power peak at 20 Hz. [file Image_1.TIF]
